# Pre-operative Prediction of Ki-67 Expression in Various Histological Subtypes of Lung Adenocarcinoma Based on CT Radiomic Features

**DOI:** 10.3389/fsurg.2021.736737

**Published:** 2021-10-18

**Authors:** Zhiwei Huang, Mo Lyu, Zhu Ai, Yirong Chen, Yuying Liang, Zhiming Xiang

**Affiliations:** ^1^Graduate School, Guangzhou University of Chinese Medicine, Guangzhou, China; ^2^Department of Radiology, Guangzhou Panyu Central Hospital, Guangzhou, China; ^3^School of Life Sciences, South China Normal University, Guangzhou, China

**Keywords:** lung adenocarcinoma, Ki-67, computed tomography, radiomics, pre-operative prediction, non-invasive biomarker

## Abstract

**Purpose:** The aims of this study were to combine CT images with Ki-67 expression to distinguish various subtypes of lung adenocarcinoma and to pre-operatively predict the Ki-67 expression level based on CT radiomic features.

**Methods:** Data from 215 patients with 237 pathologically proven lung adenocarcinoma lesions who underwent CT and immunohistochemical Ki-67 from January 2019 to April 2021 were retrospectively analyzed. The receiver operating curve (ROC) identified the Ki-67 cut-off value for differentiating subtypes of lung adenocarcinoma. A chi-square test or *t*-test analyzed the differences in the CT images between the negative expression group (*n* = 132) and the positive expression group (*n* = 105), and then the risk factors affecting the expression level of Ki-67 were evaluated. Patients were randomly divided into a training dataset (*n* = 165) and a validation dataset (*n* = 72) in a ratio of 7:3. A total of 1,316 quantitative radiomic features were extracted from the Analysis Kinetics (A.K.) software. Radiomic feature selection and radiomic classifier were generated through a least absolute shrinkage and selection operator (LASSO) regression and logistic regression analysis model. The predictive capacity of the radiomic classifiers for the Ki-67 levels was investigated through the ROC curves in the training and testing groups.

**Results:** The cut-off value of the Ki-67 to distinguish subtypes of lung adenocarcinoma was 5%. A comparison of clinical data and imaging features between the two groups showed that histopathological subtypes and air bronchograms could be used as risk factors to evaluate the expression of Ki-67 in lung adenocarcinoma (*p* = 0.005, *p* = 0.045, respectively). Through radiomic feature selection, eight top-class features constructed the radiomic model to pre-operatively predict the expression of Ki-67, and the area under the ROC curves of the training group and the testing group were 0.871 and 0.8, respectively.

**Conclusion:** Ki-67 expression level with a cut-off value of 5% could be used to differentiate non-invasive lung adenocarcinomas from invasive lung adenocarcinomas. It is feasible and reliable to pre-operatively predict the expression level of Ki-67 in lung adenocarcinomas based on CT radiomic features, as a non-invasive biomarker to predict the degree of malignant invasion of lung adenocarcinoma, and to evaluate the prognosis of the tumor.

## Introduction

Lung adenocarcinoma is the most commonly diagnosed histological subtype of non-small-cell lung cancer (NSCLC), which is the leading cause of cancer-related deaths worldwide (1). In 2011, a new classification system for lung adenocarcinomas according to the International Association for the study of Lung Cancer (IASLC), American Thoracic Society (ATS), and European Respiratory Society (ERS) has been put forward, wherein the lung adenocarcinomas are mainly classified as atypical adenomatous hyperplasia (AAH), adenocarcinoma *in situ* (AIS), minimally invasive adenocarcinoma (MIA), and invasive adenocarcinoma (IAC). Among them, AAH and AIS were pre-invasive lesions (2). More and more treatment methods can be used for the treatment of lung cancer. However, many patients, even patients with resectable lung cancer, still have poor prognoses (3). For lung adenocarcinoma, studies have found that, even for patients with complete surgical resection and in pathologic stage T1 (pathologic-T1, pT1), the treatment effects and prognoses may be significantly different (4). There is an urgent need to determine reliable prognostic factors that can predict clinical outcomes and more precisely stratify the group of patients susceptible to poorer outcomes.

Currently, Ki-67 is commonly regarded as a prognosis biomarker to predict the cell proliferation and aggressiveness of tumors in clinical practice, which can be used for quantitative analysis of tumor growth fraction and the classification of tumors and for assisting in early diagnosis and therapeutic effect evaluations (5). Ki-67 is expressed at all stages of the cell cycle except G0, with the highest expression levels in the G2/M phase. It has been reported that the overall survival (OS) and disease-free survival (DFS) of patients with high Ki-67 expression are shorter than those with low Ki-67 expression (5–7). Previous studies have identified the Ki-67 labeling index as a strong prognostic biomarker for lung adenocarcinoma (8, 9). Yamashita et al. found that Ki-67 can be used as an indicator of recurrence of lung cancer after resection (10), and the level of its positive expression is closely related to the differentiation degree, lymph node metastasis, and other factors of lung cancer (8, 11). The most commonly used method to quantify Ki-67 expression is immunohistochemistry (IHC), which is not practical for the dynamic monitoring of Ki-67 during lung cancer treatment because of invasion, which is time-consuming (12). Due to the existence of tumor heterogeneity, Ki-67 values varied in different regions of the tumor samples, and traditional invasive immunohistochemical methods only evaluate the biopsy specimens of a small sample of the tissue and cannot reflect the overall heterogeneity of the tumor (13, 14). Therefore, finding a non-invasive, cost-effective, and comprehensive method for clinical Ki-67 expression level assessment is necessary.

Radiomics is a recently emerging technique in computational medical imaging. It involves the extraction and analyses of a large number of quantitative imaging features from medical images (15, 16). It is different from traditional methods because it converts medical images into mineable high-dimensional data. Radiomics can help support patient diagnosis, prognosis, treatment, and prediction in clinical practice. The relationship between Ki-67 expression level and radiomic features has always been a hot topic. Studies have shown that radiomics can be used to pre-operatively predict the expression level of Ki-67 in breast cancer (17) and adrenal cancer (18). In addition, several studies have shown that the quantitative imaging features from CT can predict Ki-67 levels and subtypes in patients with lung cancer (19, 20), but the role of Ki-67 in distinguishing the pathological stages of lung adenocarcinoma remains unclear (12, 21). To our knowledge, there have been no studies on the use of CT-based radiomic features to predict Ki-67 expression levels in subtypes of lung adenocarcinoma.

This study aimed to investigate the correlation between Ki-67 expression level and the subtypes of lung adenocarcinoma and to assess whether CT-based radiomic features could serve as non-invasive predictors of the Ki-67 levels in patients with lung adenocarcinoma.

## Materials and Methods

### Patient Characteristics

We retrospectively collected data from patients who underwent a chest CT scan, Ki-67 expression level detection, and post-operative pathological confirmation of lung adenocarcinoma at our institute from January 2019 to April 2021. The inclusion criteria were: (1) patients confirmed with lung adenocarcinoma by surgical resection, (2) maximum diameter of tumor ≤ 3 cm, (3) complete clinicopathological data, (4) IHC examination of Ki-67 expression levels, and (5) complete CT images. The exclusion criteria were: (1) greatest tumor diameter >3 cm, (2) radiotherapy, chemotherapy, or radiotherapy and chemotherapy were performed before surgery, and (3) incomplete or poor-quality CT images. Our institutional review board approved this retrospective study, and the requirement for informed consent was waived.

### Computed Tomography Examination

All patients in this study used a 64-slice CT scanner (Discovery CT 750 HD, GE Healthcare, Chicago, IL, USA) for their chest scans. All CT scans were obtained with the patients in the supine position and holding their breath at the end of a full inspiration. The scan ranged from above the apex of the lungs to below the level of the diaphragm. The scanning parameters were as follows: tube voltage of 120 or 140 kV, tube current of 200–340 mA, beam pitch of 1.2, pixel resolution of 512 ×512, field of view (FOV) of 360 mm, thickness of 5 mm, and reconstructed slice thickness and slice increment of 1 mm. Afterward, the CT scans were reviewed as lung window images (window width = 1,200 HU; window level = −700 HU) and mediastinal window images (window width = 350 HU; window level = 50 HU). All images were exported in a DICOM format for image feature extraction after scanning.

The CT imaging signs included (22): (1) lesion location, (2) maximum diameter of the tumor on axial images, (3) tumor-lung interface: clear or unclear, (4) density: pure ground-glass opacity, mixed ground-glass opacity, and solid nodule, (5) spiculation, (6) lobulation, (7) bubble-like lucency, (8) air bronchogram sign, (9) vascular sign, and (10) pleural traction. Two diagnostic radiologists with 3 and 9 years of experience reviewed the CT images of each patient and identified positive and negative findings by consensus. The entire process was performed without the patient having knowledge of the pathological results.

### Immunohistochemical Analysis

Lung tissues were fixed with a 10% buffered formaldehyde solution by transbronchial or transpleural perfusion for ~48 h and embedded in paraffin wax. Tissue sections were stained with HE. A mouse anti-human Ki-67 monoclonal antibody (Beijing Zhongshan Jinqiao Biotechnology Co., Ltd., Beijing, China) was used to perform the immunohistochemical detection according to the kit instructions. Positive and negative controls were set up, respectively. Ki-67 was positive with brown-yellow granules in the nucleus. The number of Ki-67 positive tumor cells was calculated in five fields of high-power field (×400) under light microscopes. The percentage of Ki-67 expression level positive staining of tumor cells in each field = the number of positive tumor cells in each field/total tumor cells in each field × 100%. The Ki-67 indices in five fields were calculated and averaged. Histological and cytological subtypes were assessed according to the WHO classification system for lung cancer (5th version) (23). The thresholding of the Ki-67 expression level was used to separate the tumor samples into positive and negative groups: The expression level of Ki-67 ≤ 5% was negative, and >5% was positive (24).

### Radiomics Analysis

#### Image Pre-processing and Image Segmentation

Firstly, the CT scan images of all patients were exported in a DICOM format from the PACS system workstations, and the AK (Analysis Kinetics, V3.2.0, Workbench2014, GE Healthcare) software was used to preprocess the resampled images of 0.5 × 0.5 × 0.5 on the X, Y, and Z axes, respectively. The three-dimensional segmentation of the tumor regions of interest (ROIs) was performed using the ITK-SNAP software (version 3.8, Philadelphia, PA, USA) with the window width and window level as 1,200 HU and −700 HU, respectively. Then, the ROIs were outlined, and the outlined image was saved in the format of “Merge. nii.” The scope of the image delineation includes tumor necrosis, cystic, and cavity, excluding burr, thickened pleura, and surrounding signs. The continuous delineation includes the whole lesion. If it is found to be contradictory, other senior radiologists will evaluate the tumor mask again to reach an agreement.

#### Radiomic Feature Extraction

The data were imported into the AK (V3.2.0, Workbench2014, GE Healthcare) analysis software to extract radiomic features, including features of first order, shape (Shape), gray-level co-occurrence matrix (GLCM), gray-level run-length matrix (GLRLM), gray-level size-zone matrix (GLSZM), gray-level dependence matrix (GLDM), and features of neighborhood gray difference matrix (NGTDM). The selected image transformations were: logarithmic transformation (LoG), parameter Sigma selection 2.0, 3.0; wavelet transformation (Wavelet), Level 1; local binary mode (LBP), Level 2, Radius 1.0, Subdivision select 1. A total of 1,316 features were extracted.

#### Radiomic Feature Selection and Classifier Construction

**Figure 1:**
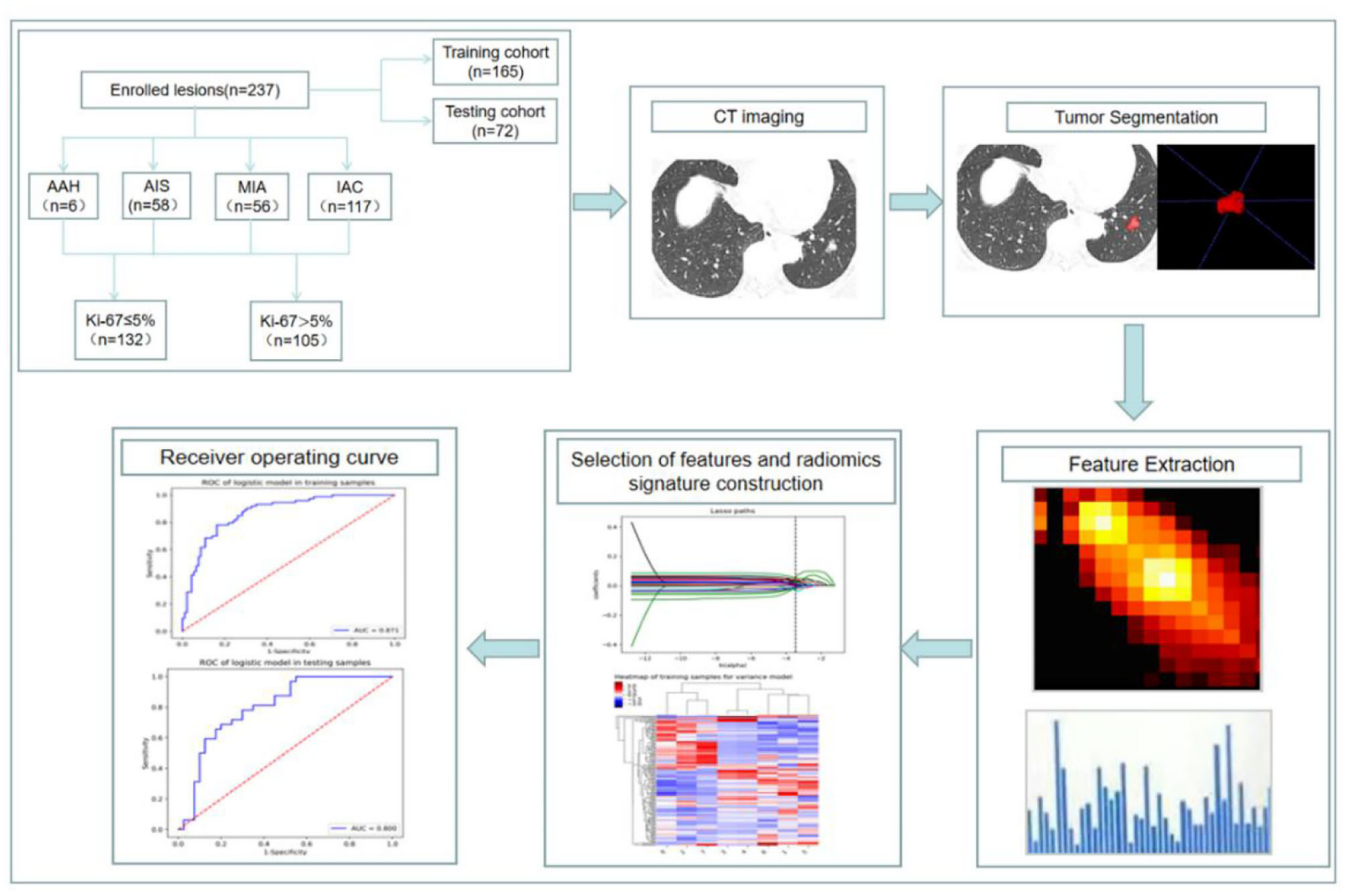
Flowchart of constructing a CT radiomic feature model to predict the Ki-67 expression level in lung adenocarcinoma.

The interobserver intraclass correlation coefficient (ICC) selects values >0.75. Stratified sampling was used to divide all the patients into a training cohort (*n* = 165) and a validation cohort (*n* = 72) according to a ratio of 7:3. First, an ANOVA was performed to remove features with *p* > 0.05, and then the rest of the radiomic features were retained to select the most relevant features using recursive feature elimination (RFE). Next, the least absolute shrinkage and selection operator (LASSO) model, which could improve prediction accuracy and interpretation, was used to further select the features. According to Mann–Whitney *U* test, the top-class features were screened out to build the final logistic regression classifier, which was used to perform radiomic feature selection in the training dataset. Classification performance was evaluated using the area under the receiver operating characteristic curve (AUC). Finally, a radiomic score (Rad score) was developed using the logistic regression model and then used to calculate the training and validation datasets. A simplified flowchart of the study is given in Figure 1.

### Statistical Analysis

#### Clinicopathologic Characteristics of Patients

All statistical analyses were performed with SPSS version 21.0 (IBM Corporation, Armonk, NY, USA). Results were given as mean ± SD or median and range values. The chi-square test or Fisher's exact test was adopted to compare the distribution of the categorical variables. Student's *t*-test or one-way ANOVA was also calculated for the comparison of continuous variables. Manne–Whitney *U* testing was used for non-parametric data. Binary logistic regression was used to analyze the potential risk factors affecting the Ki-67 expression level. The cut-off value of the labeling index was obtained from the receiver operating curve (ROC) with the Youden index. The statistical analysis was considered significant when the *p*-value was <0.05.

#### Performance of the Radiomic Prediction Model

To evaluate the performance of the proposed radiomic prediction model, we adopted accuracy, sensitivity, specificity, positive predictive value, and negative predictive value as the evaluation indexes. Furthermore, the ROC curves and the AUCs were calculated to quantitatively assess the predictive capacity of the radiomic classifiers in the training and validation datasets. A *p*-value of <0.05 was considered statistically significant.

## Results

### Characteristics of Study Subjects

The clinicopathologic characteristics of the patients with lung adenocarcinoma were summarized in Table 1. After screening, a total of 215 patients met the requirements. Among them, 19 patients had multiple lesions. There were 68 males (31.63%) and 147 females (68.37%) with a median age of 56 years old. One hundred ninety-six patients (91.16%) were non-smokers. Seventeen patients (7.91%) had a history of non-pulmonary tumors, 9 had thyroid cancer, 4 had breast cancer, 1 had cervical cancer, 1 had endometrial squamous cell carcinoma, 1 had colon cancer, and 1 had vocal cord squamous cell carcinoma. In the end, 237 lung adenocarcinoma lesions were selected for our study.

**Table 1:** Patient characteristics on a per patient level.

**Variables**	***n*** **(%) or Median (range)**
Age (years)	56.00 (22,82)
**Gender**	
Female	147 (68.37)
Male	68 (31.63)
**Smoking history**	
Never	196 (91.16)
Ever	19 (8.84)
History of malignancies	17 (7.91)
Breast cancer	4 (1.86)
Cervical cancer	1 (0.47)
Thyroid cancer	9 (4.19)
Colon cancer	1 (0.47)
Endometrial squamous cell carcinoma	1 (0.47)
Vocal cord squamous cell carcinoma	1 (0.47)

### Ki-67 Expression Levels and Histological Subtypes

**Figure 2:**
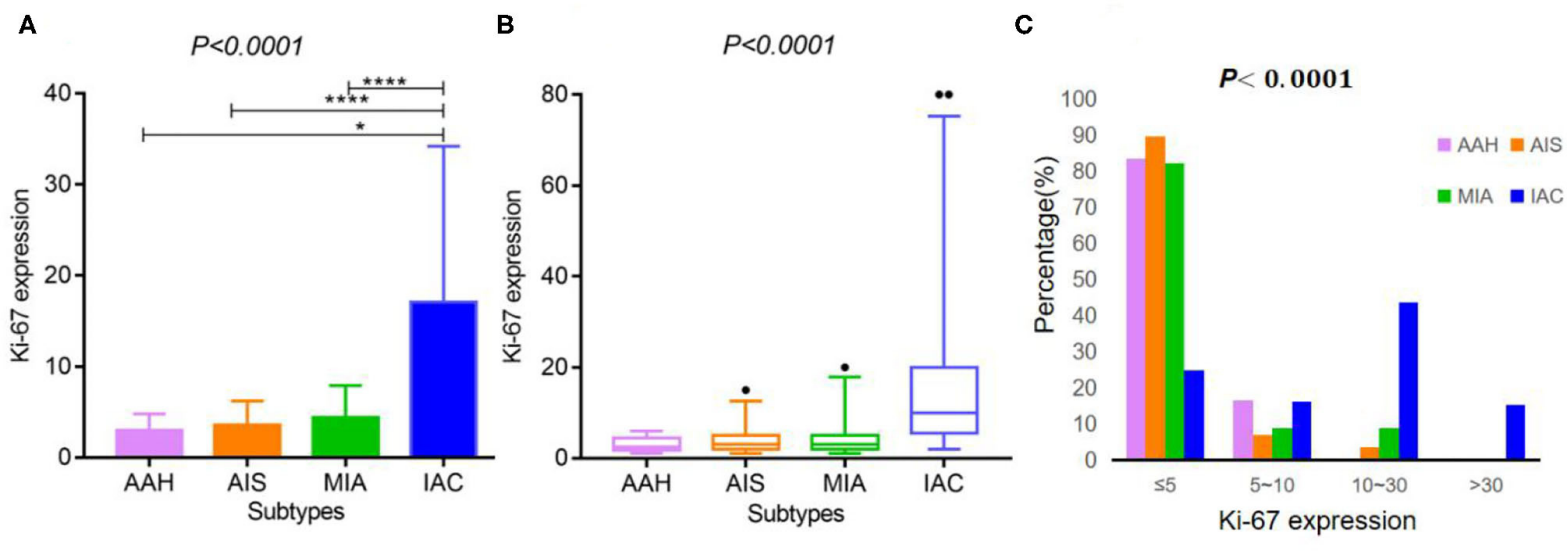
Ki-67 expression according to the histological subtypes of lung adenocarcinoma. **(A,B)** Ki-67 expression differed across various lung adenocarcinoma subtypes [**A**: mean ± SD, **B**: median (2.5–97.5%)]. **(C)** The expression distribution of Ki-67 according to the histological subtypes of lung adenocarcinoma. **** indicates *P*-value <0.01, and * indicates *P*-value <0.05.

As shown in Tables 2, 3, and Figures 2A–C, the pathological diagnoses based on multidisciplinary lung adenocarcinoma criteria were as follows: 6 patients (2.53%) had AAH, 58 patients (24.47%) had AIS, 56 patients (23.63%) had MIA, and 117 (49.37%) had IAC. The AAH subtype had the lowest Ki-67 expression level (3 ± 1.79), followed by AIS (3.57 ± 2.63), MIA (4.39 ± 3.53), and IAC (17.09 ± 17.12). The samples were further divided into four subgroups according to Ki-67 expression: ≤ 5, 5–10, 10–30, and >30%. In total, the group with Ki-67 expression levels ≤ 5% was composed of pre-cellular (AAH/AIS) (57/64, 89.06%), minimally invasive (46/56, 82.14%), and invasive adenocarcinomas (29/117, 24.79%). The group with Ki-67 expression levels >5% consisted of pre-cellular (7/64, 10.94%), minimally invasive (10/56, 17.86%), and invasive adenocarcinomas (88/117, 75.21%). By a one-way ANOVA, Tamhane's T2 test (with a Levene test for uneven variance between groups) compared the expression levels of Ki-67, and it was found that there was no significant difference between the AAH group and AIS group (*p* = 0.608), the AAH group and MIA group (*p* = 0.347), and the AIS group and MIA group (*p* = 0.159), but the AAH group (*p* <0.001), AIS group (*P* <0.001), and MIA group (*p* <0.001) were significantly different from the IAC group.

**Table 2:** The expression level of Ki-67 in various pathological subtypes of lung adenocarcinoma.

**Variables**	**AAH (***n*** = 6)**	**AIS (***n*** = 58)**	**MIA (***n*** = 56)**	**Non-invasive adenocarcinoma (AAH/AIS/MIA) (***n*** = 120)**	**IAC (***n*** = 117)**	***P***
**Ki-67 expression (%)**						
mean ± SD	3.00 ± 1.79	3.57 ± 2.63	4.39 ± 3.53	3.93 ± 3.07	17.09 ± 17.12	<0.001
Median (range)	2.5 (1,6)	3 (1,15)	3 (1,20)	3 (1,20)	10 (2,80)	<0.001
**Ki-67 Subgroups (%)**						<0.001
≤ 5	5 (83.33)	52 (89.65)	46 (82.14)	103 (85.83)	29 (24.79)	
5–10	1 (16.67)	4 (6.90)	5 (8.93)	10 (8.34)	19 (16.24)	
10–30	0	2 (3.45)	5 (8.93)	7 (5.83)	51 (43.59)	
>30	0	0	0	0	18 (15.38)	

*SD, standard deviation*.

**Table 3:** Area under the ROC curve (AUC) of the Ki-67 expression level among different pathological subtypes paired groups.

**Pairs**	***P***	**AUC**	**Optional cut-off value**	**Standard deviation**	**Asymptotic *P*-value**	**Asymptotic 95% Confidence interval**	
						**Lower limit**	**Upper limit**
MIA vs. IAC	<0.001	0.851	5.5	0.030	<0.001	0.792	0.909
AAH/AIS vs. MIA/IAC	<0.001	0.787	6.5	0.030	<0.001	0.729	0.846
AAH/AIS/MIA vs. IAC	<0.001	0.872	5.5	0.023	<0.001	0.828	0.916

*AAH, atypical adenomatous hyperplasia; AIS, adenocarcinoma in situ; MIA, minimally invasive adenocarcinoma; IAC, invasive adenocarcinoma*.

### The Optimal Ki-67 Cut-Off Points Among Different Pathological Subtypes Paired Groups of Lung Adenocarcinoma

**Figure 3:**
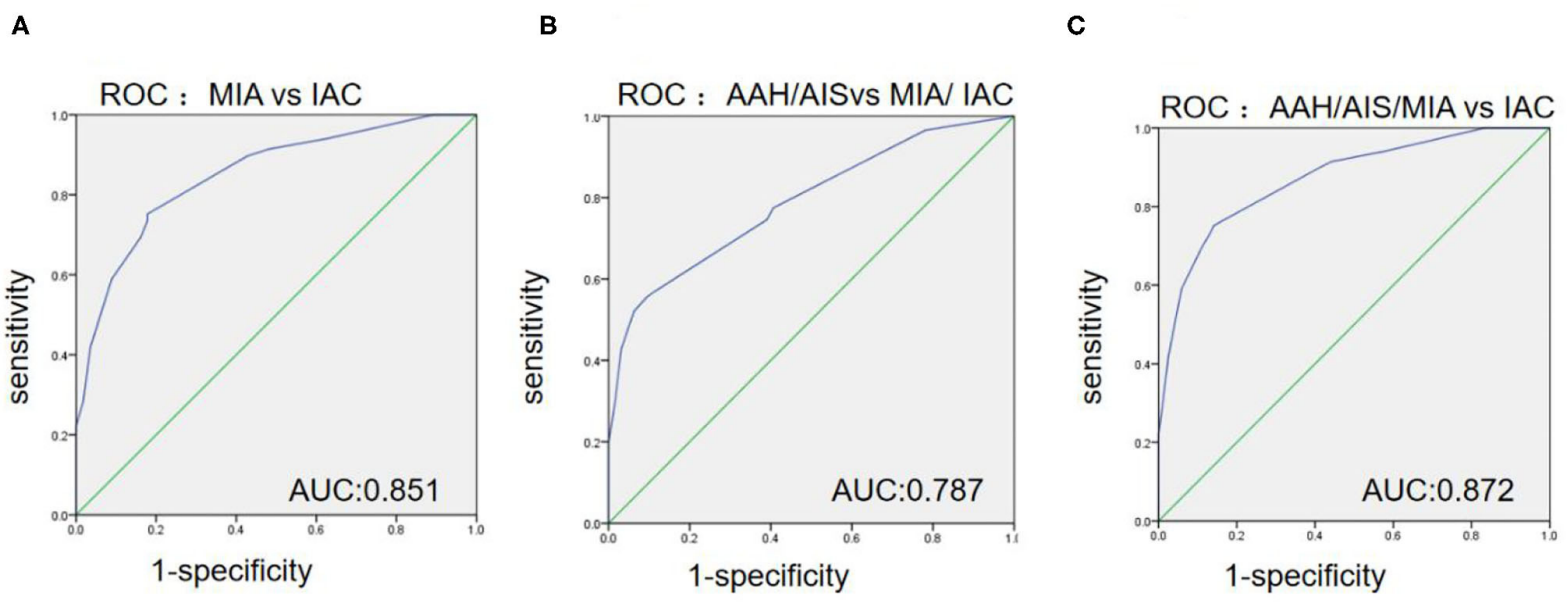
The ROC curves among the different paired groups of pathological subtypes. **(A)** Minimally invasive adenocarcinoma (MIA) vs. invasive adenocarcinoma (IAC); **(B)** Atypical adenomatous hyperplasia (AAH)/adenocarcinoma *in situ* (AIS) vs. MIA/IAC; **(C)** AAH/AIS/MIA vs. IAC.

As shown in Table 3 and Figures 3A–C, the ROC curve was used to analyze the sensitivity, specificity, and cut-off value of Ki-67 as a discriminant index for different pathological subtypes of lung adenocarcinoma. Non-invasive lung adenocarcinoma (AAH/AIS/MIA) vs. invasive lung adenocarcinoma (AUC = 0.872) had the highest sensitivity and specificity, followed by MIA vs. IAC (AUC = 0.851) and pre-cellular (AAH/AIS) vs. MIA/IAC (AUC = 0.787) (*p* <0.001). According to the cut-off points of each pairing group, when the expression of Ki-67 was ≤ 5%, it was more inclined to AAH, AIS, or MIA, while more than 5% corresponded to IAC.

### Comparison of CT Imaging Signs and Clinical Data of Lung Adenocarcinoma in Ki-67 Negative and Positive Expression Group

**Figure 4:**
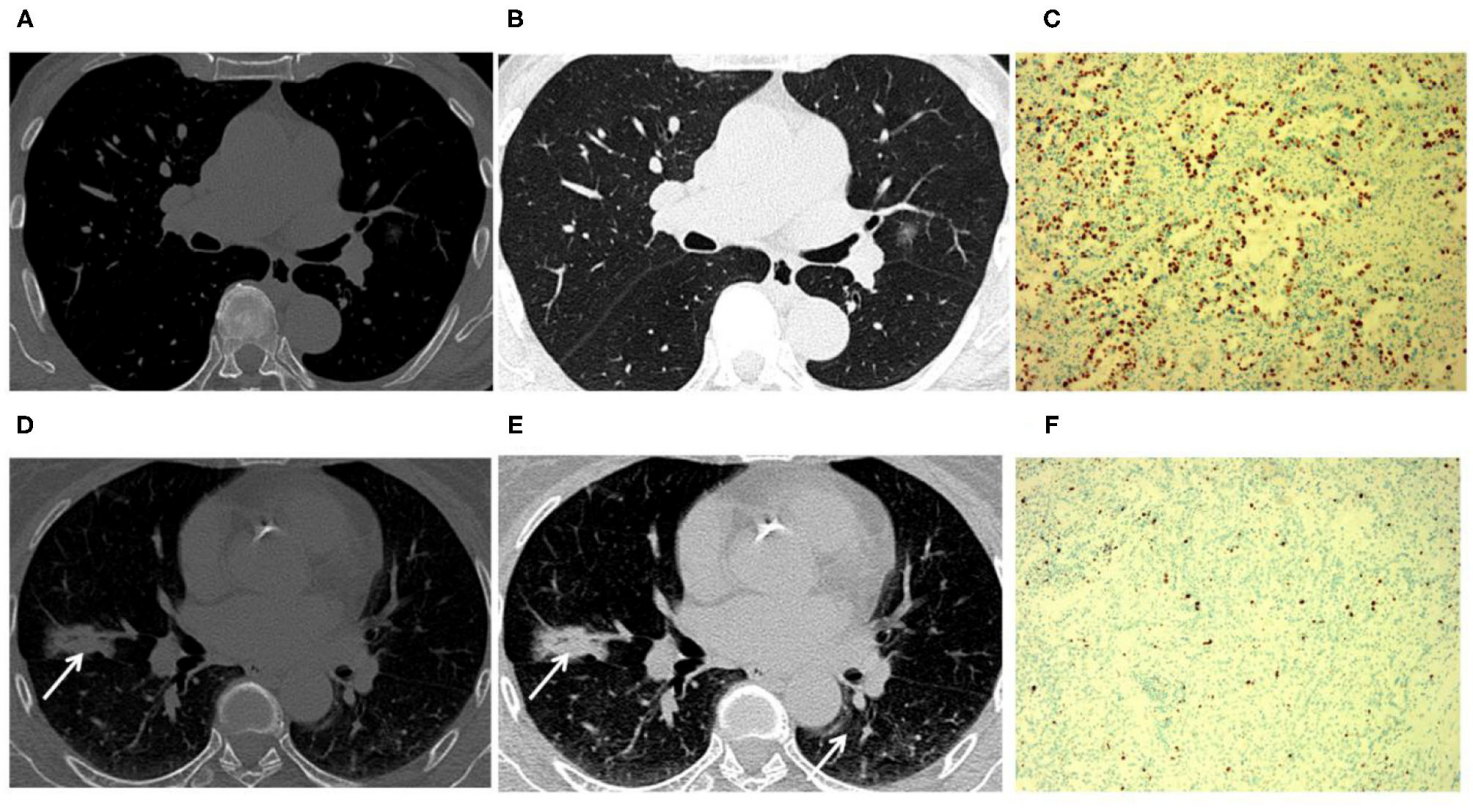
Tumors with positive Ki-67 expression levels were more inclined to have air bronchograms than those with negative Ki-67 expression levels. An 81-year-old male patient with Ki-67 negative expression **(A–C)**. **(A,B)** CT image showed that a tumor had a negative expression of Ki-67 without an air bronchogram. **(C)** The Ki-67 expression was 5% in the glandular epithelium. A 63-year-old female patient with positive Ki-67 expression **(D–F)**. **(D,E)** CT image showed that a tumor had positive Ki-67 expression with an air bronchogram. **(F)** The Ki-67 expression was 55% in the glandular epithelium (arrow).

In our study, the median expression of Ki-67 was 5%. In addition, the lung adenocarcinomas were divided into a non-invasive adenocarcinoma group (AAH/AIS/MIA group) and an invasive adenocarcinoma group according to the prognosis of the lesions. Those classified as MIA were grouped with AAH/AIS due to its good prognosis. Therefore, 5% was selected as the cut-off value for grouping different stages of lung adenocarcinoma. Patients were divided into two groups: 132 (55.7%) patients had negative Ki-67 expression, and 105 (44.3%) patients exhibited positive Ki-67 expression. We first explored whether the imaging signs and clinical data could distinguish between the Ki-67 negative expression group and the Ki-67 positive expression group. The results showed that CT imaging signs (maximum diameter, density, shape, lobulation, spiculation, air bronchogram, vascular sign, and pleural traction) could be used to discriminate between the two groups (*p* <0.001). There was a higher percentage of lymph node metastasis in the Ki-67 positive expression group than in the Ki-67 negative expression group (*p* < 0.001) (Table 4). Air bronchogram and histopathological subtype had moderate predictive values, and the AUC values were 0.711 and 0.809, respectively (Table 5). The Ki-67 positive expression group was more inclined to have air bronchograms than the Ki-67 negative expression group. The histopathological subtype of the Ki-67 positive expression group was more likely to be IAC, while the Ki-67 negative expression group was more likely to be a pre-invasive lesion (AAH and AIS) or MIA (Figure 4).

**Table 4:** Comparison of imaging features and histopathological subtypes in the Ki-67 negative and positive expression groups of lung adenocarcinoma.

**Characteristics**	**Negative Ki-67 expression group (*****n*** **=** **132)**		**Total**	**Positive Ki-67 expression group (*****n*** **=** **105)**		**Total**	***p***
	**Training cohort**	**Testing cohort**		**Training cohort**	**Testing cohort**		
	**(***n*** = 92)**	**(***n*** = 40)**		**(***n*** = 73)**	**(***n*** = 32)**		
**Pathological subtype**							<0.001
Pre-cellular (AAH/AIS)	38 (28.79)	19 (14.39)	57 (43.18)	4 (3.81)	3 (2.86)	7 (6.67)	
MIA	35 (26.52)	11 (8.33)	46 (34.85)	7 (6.67)	3 (2.86)	10 (9.52)	
IAC	19 (14.39)	10 (7.58)	29 (21.97)	62 (59.05)	26 (24.76)	88 (83.81)	
LPA	1 (0.76)	4 (3.03)	5 (3.79)	11 (10.48)	1 (0.95)	12 (11.43)	
APA	15 (11.36)	4 (3.03)	19 (14.39)	39 (37.14)	19 (18.10)	58 (55.24)	
PPA	2 (1.52)	1 (0.76)	3 (2.27)	7 (6.67)	3 (2.86)	10 (9.52)	
MPA	0	0	0	2 (1.90)	1 (0.95)	3 (2.86)	
SPA	1 (0.76)	1 (0.76)	2 (1.52)	3 (2.86)	2 (1.90)	5 (4.76)	
**Lymph node metastasis**							<0.001
Yes	0	0	0	10 (9.52)	4 (3.81)	14 (13.33)	
No	92 (69.70)	40 (30.30)	132 (100.00)	63 (60.00)	28 (26.67)	91 (86.67)	
Maximum diameter	7.20 ± 4.30	8.63 ± 5.12	7.63 ± 4.59	14.34 ± 5.82	15.19 ± 6.42	14.60 ± 5.99	<0.001
**Lesion location**							0.923
Right upper lobe	34 (25.76)	12 (9.09)	46 (34.85)	24 (22.86)	12 (11.43)	36 (34.29)	
Right middle lobe	6 (4.55)	0	6 (4.55)	1 (0.95)	2 (1.90)	3 (2.86)	
Right lower lobe	20 (15.15)	11 (8.33)	31 (23.48)	16 (15.24)	7 (6.67)	23 (21.90)	
Left upper lobe	23 (17.42)	9 (6.82)	32 (24.24)	17 (16.19)	9 (8.57)	26 (24.76)	
Left lower lobe	9 (6.82)	8 (6.06)	17 (12.88)	15 (14.29)	2 (1.90)	17 (16.19)	
**Density**							<0.001
pGGO	51 (38.64)	21 (15.91)	72 (54.55)	8 (7.62)	1 (0.95)	9 (8.57)	
mGGO	36 (27.27)	15 (11.36)	51 (38.64)	38 (36.19)	20 (19.05)	58 (55.24)	
SN	5 (3.79)	4 (3.03)	9 (6.82)	27 (25.71)	11 (10.48)	38 (36.19)	
**Shape**							<0.001
Round	70 (53.03)	30 (22.73)	100 (75.76)	30 (28.57)	10 (9.52)	40 (38.10)	
Irregular	22 (16.67)	10 (7.58)	32 (24.24)	43 (40.95)	22 (20.95)	65 (61.90)	
**Tumor-lung interface**							0.239
Clear	47 (35.61)	19 (14.39)	66 (50.00)	44 (41.90)	17 (16.19)	61 (58.10)	
Unclear	45 (34.09)	21 (15.91)	66 (50.00)	29 (27.62)	15 (14.29)	44 (41.90)	
**Lobulation**							<0.001
Yes	58 (43.94)	26 (19.70)	84 (63.64)	69 (65.71)	30 (28.57)	99 (94.29)	
No	34 (25.76)	14 (10.61)	48 (36.36)	4 (3.81)	2 (1.90)	6 (5.71)	
**Spiculation**							<0.001
Yes	35 (26.52)	14 (10.61)	49 (37.12)	56 (53.33)	26 (24.76)	82 (78.10)	
No	57 (43.18)	26 (19.70)	83 (62.88)	17 (16.19)	6 (5.71)	23 (21.90)	
**Bubblelike lucency**							0.418
Yes	35 (26.52)	16 (12.12)	51 (38.64)	22 (20.95)	13 (12.38)	35 (33.33)	
No	57 (43.18)	24 (18.18)	81 (61.36)	51 (48.57)	19 (18.10)	70 (66.67)	
**Air bronchogram**							<0.001
Yes	33 (25.00)	13 (9.85)	46 (34.85)	54 (51.43)	23 (21.90)	77 (73.33)	
No	59 (44.70)	27 (20.45)	86 (65.15)	19 (18.10)	9 (8.57)	28 (26.67)	
**Vascular sign**							<0.001
Yes	72 (54.55)	33 (25.00)	105 (79.55)	72 (68.57)	30 (28.57)	102 (97.14)	
No	20 (15.15)	7 (5.30)	27 (20.45)	1 (0.95)	2 (1.90)	3 (2.86)	
**Pleural traction**							<0.001
Yes	32 (24.24)	11 (8.33)	43 (32.58)	48 (45.71)	20 (19.05)	68 (64.76)	
No	60 (45.45)	29 (21.97)	89 (67.42)	25 (23.81)	12 (11.43)	37 (35.24)	

*pGGO, pure ground-glass opacity; mGGO, mixed ground-glass opacity; SN, solid nodule; AAH, atypical adenomatous hyperplasia; AIS, adenocarcinoma in situ; MIA, minimally invasive adenocarcinoma; IAC, invasive adenocarcinoma; LPA, lepidic-predominant adenocarcinoma; APA, acinar-predominant adenocarcinoma; PPA, papillary-predominant adenocarcinoma; MPA, micropapillary-predominant adenocarcinoma; SPA, solid-predominant adenocarcinoma*.

**Table 5:** ROC curve of the main factors affecting the Ki-67 expression level.

**Characteristics**	**AUC**	**B**	**S.E**.	**Wals**	**Exp(B)**	**Exp(B)**		***P***
						**Low limit**	**Up limit**	
Pathological subtype	0.809	−1.459	0.519	7.90	0.232	0.084	0.643	0.005
Air bronchogram sign	0.711	−0.813	0.406	4.014	0.444	0.200	0.982	0.045

*AUC, area under curve; S.E., standard error; Exp, exponential function*.

### Feature Selection

**Figure 5:**
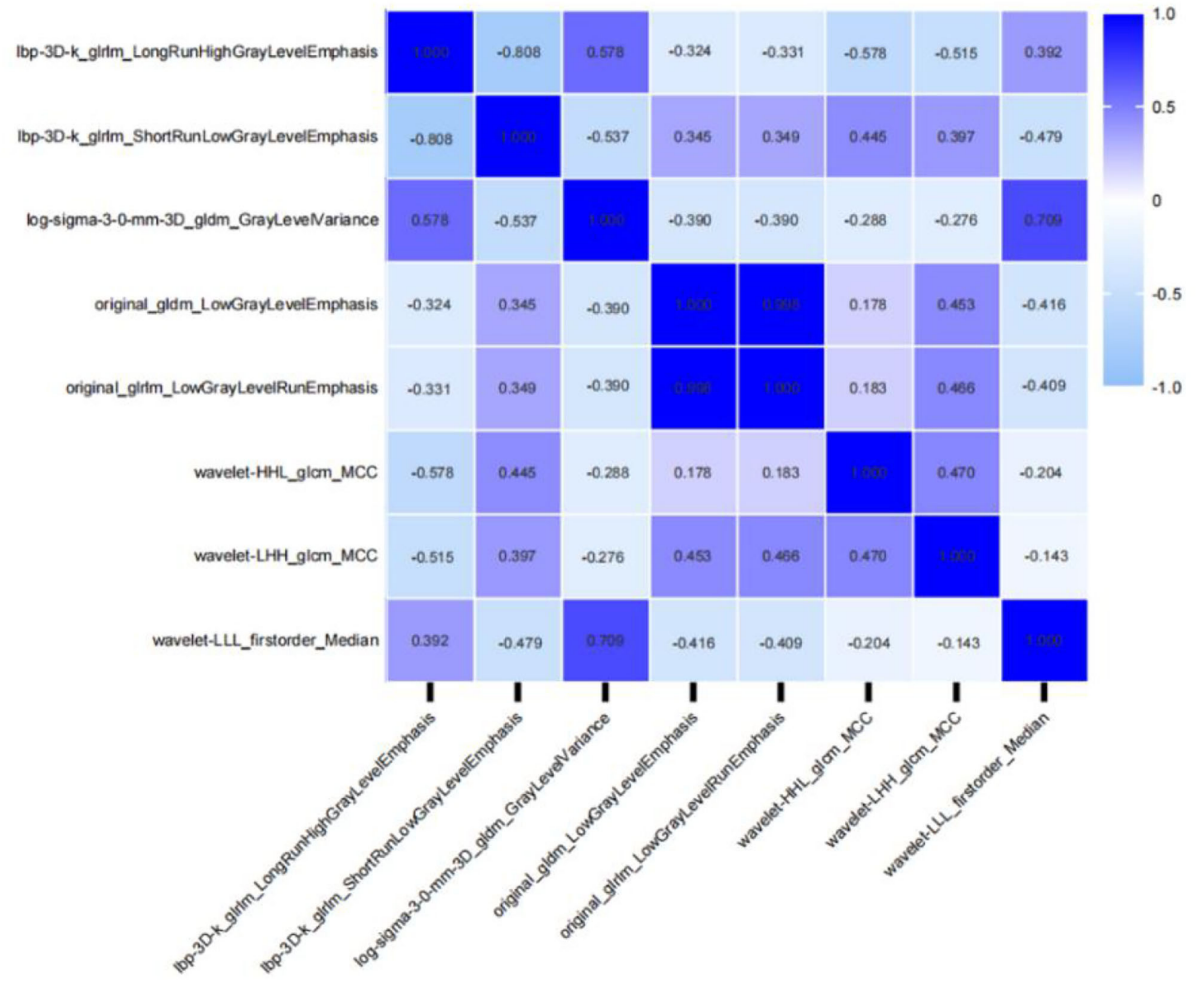
Correlative heatmap between eight top-class radiomic features and Ki-67 levels. The values in the square lattices represent the magnitude of the R-value of a correlation analysis displayed by color difference.

Patients were randomly divided into a training dataset (*n* = 165) and a validation dataset (*n* = 72) in a ratio of 7:3. At last, 1,316 radiomic features were extracted from the AK software. After the application of LASSO logistic algorithm, eight radiomic features were finally selected as the optimal radiomic feature subset based on the relationship between the classification accuracy and the number of features for the radiomic classifier. In Table 6, eight optimal radiomic features include 1 first order histogram feature, 2 GLCM,3 GLRLM, and 2 GLDM. Figure 5 shows the correlation between the top eight features, namely, lbp-3D-k_glrlm_LongRunHighGrayLevelEmphasis (*p* <0.001), lbp-3D-k_glrlm_ShortRunLowGrayLevelEmphasis (*p* <0.001), log-sigma-3-0-mm-3D_gldm _GrayLevelVariance (*p* < 0.001), original_gldm_LowGrayLevelEmphasis (*p* < 0.001), original_glrlm_LowGrayLevelRunEmphasis (*p* < 0.001), wavelet-HHL_glcm_MCC (*p* < 0.001), wavelet-LHH_glcm_MCC (*p* < 0.001),and wavelet-LLL_firstorder_Median (*p* < 0.001).

**Table 6:** Analysis of the radiomic features between negative and positive Ki-67 levels in the training set.

**Radiomic features**	**Ki-67 negative expression group (***n*** = 92)**	**Ki-67 positive expression group (***n*** = 73)**	***p***
	**Mean (range)**	**Mean (range)**	
lbp-3D-k_glrlm_LongRunHighGrayLevelEmphasis	−0.3747 (−2.55, 1.88)	0.4722 (−0.89, 5.79)	<0.001
lbp-3D-k_glrlm_ShortRunLowGrayLevelEmphasis	0.3820 (−1.68, 6.27)	−0.4814 (−0.3279, −2.36)	<0.001
log-sigma-3-0-mm-3D_gldm_GrayLevelVariance	−5.124 (−1.27, 1.40)	0.6458 (−1.17, 2.80)	<0.001
original_gldm_LowGrayLevelEmphasis	0.1915 (−0.70, 7.08)	−0.2414 (−0.71, 3.68)	0.004
original_glrlm_LowGrayLevelRunEmphasis	0.2163 (−0.78, 4.82)	−0.2726 (−0.80, 3.80)	0.001
wavelet-HHL_glcm_MCC	0.2603 (−2.16, 3.68)	−0.3280 (−1.70, 1.57)	<0.001
wavelet-LHH_glcm_MCC	0.2364 (−1.52, 4.38)	−0.2980 (−2.01, 3.53)	0.001
wavelet-LLL_firstorder_Median	−0.4528 (−1.13, 2.01)	0.5707 (−1.11, 2.30)	<0.001

*MCC, Maximal Correlation Coefficient*.

### Development and Validation of the Radiomic Prediction Model

**Figure 6:**
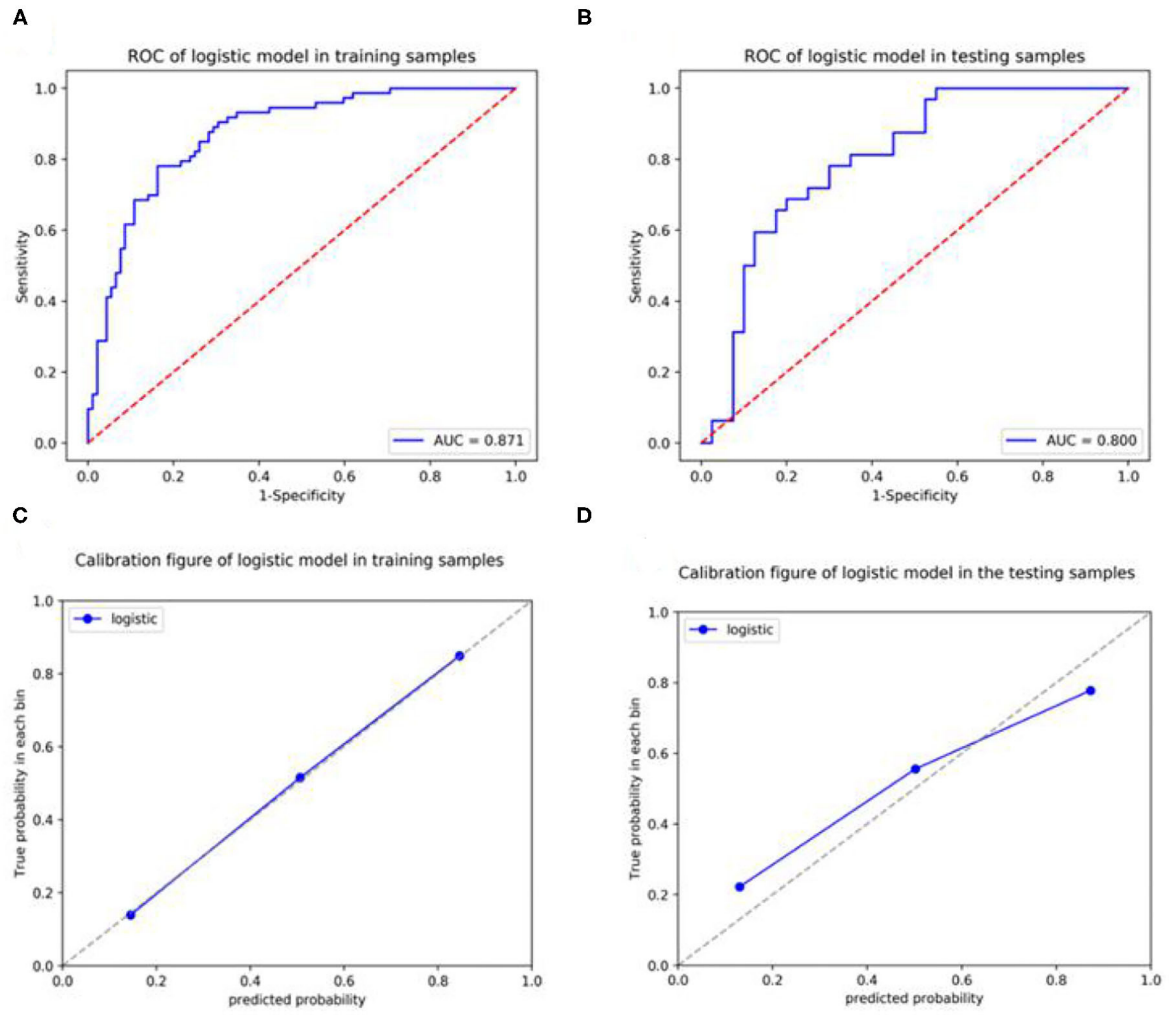
Receiver operating curves **(**ROCs) and a calibration curve analysis for the radiomic signature for predicting the expression level of Ki-67 in the training set and testing set. [**(A,C)** training dataset, **(B,D)** testing dataset]. Calibration curves depict the calibration of each model in terms of the agreement between the predicted and actual probability of the positive Ki-67 expression level rate. The Y-axis represents the actual rate. The X-axis represents the predicted probability. The diagonal dotted line represents perfect prediction by an ideal model. The blue line represents the performance of the radiomic prediction model, of which a closer fit to the diagonal blue dotted line represents a better prediction.

In the training set, the expression level of Ki-67 was taken as the dependent variable, and the CT radiomic features of lung adenocarcinoma were used as the independent variable to establish a pre-operative prediction model of Ki-67 expression level. The AUC value was 0.871 in the training dataset, the sensitivity was 76.7%, and the specificity was 83.7%, with a positive predictive value of 0.789. For the testing set, the classifier had an AUC value of 0.8, the sensitivity was 68.8%, and the specificity was 80%, with a positive predictive value of 0.733 (Table 7, Figures 6A,B). The calibration curve of the radiomic features also showed that the predicted probability was in good agreement with the actual probability in the training cohort (Figures 6C,D).

**Table 7:** The predictive performance of radiomic classifier in training and validation sets.

**Datasets**	**AUC**	**SEN**	**SPE**	**Accuracy**	**PPV**	**NPV**
Training (***n*** = 165)	0.871	0.767	0.837	0.806	0.789	0.819
Testing (***n*** = 72)	0.800	0.688	0.800	0.750	0.733	0.762

*AUC, area under curve; SEN, sensitivity; SPE, specificity; PPV, positive predictive value; NPV, negative predictive value*.

## Discussion

Ki-67 is considered to represent the proliferative state of tumors and is a prognostic biomarker in multiple malignant tumors, such as breast, prostate, and lung cancer (14). Ki-67 has a broad prospect in the study of lung cancer, especially the occurrence, development, early diagnosis, and prognosis of ground-glass opacity (GGO) in early lung cancer under low-dose CT scans (25, 26). Ki-67 has been widely introduced into clinical practice to differentiate lung cancer subtypes and predict oncology outcomes (26–28). In our study, we systematically evaluated the expression level of Ki-67 according to the histological subtypes of lung adenocarcinoma and revealed the prognostic role of Ki-67 in lung adenocarcinoma. Strikingly, we found that Ki-67 expression differed across lung adenocarcinoma histological subtypes, with IAC harboring the highest expression level, followed by the MIA, AIS, and AAH subtypes, which was consistent with the finding by Ishida et al. and Yan et al. (29, 30). Ki-67 expression levels demonstrated good performance in our study, with AUCs of 0.851, 0.787, and 0.872 for differentiating between MIA and IAC, AAH/AIS and MIA/IAC, and AAH/AIS/MIA and IAC, respectively. The Youden index of the paired groups of pathological subtypes were 5.5, 6.5, and 5.5, respectively. It showed that the Ki-67 values were below 5% for non-invasive adenocarcinomas (AAH/AIS/MIA) and more than 5% for invasive adenocarcinomas. Notably, it means that Ki-67 expression could be identified as an independent prognostic factor of lung adenocarcinoma (28). The overexpression of Ki-67 infers poor differentiation and prognosis. Hence, the accurate pre-operative evaluation of the Ki-67 level may be helpful in distinguishing the different subtypes of patients with lung adenocarcinoma (31). For some suspicious patients with follow-up observations and no indication of surgery or needle biopsy, Ki-67 could serve as a useful predictive biomarker to select suspicious lesions with high proliferation. The early detection of this cancer could enhance the cure of the disease and even prolong overall survival.

Several previous studies demonstrated that conventional CT images could be a non-invasive measurement to predict the Ki-67 index in lung adenocarcinoma. Our results showed that the degree of Ki-67 expression was related to nodule diameter, density, spiculation, lobulation, and air bronchogram sign. Moreover, an air bronchogram was the independent factor influencing the Ki-67 expression level, and the AUC in the ROC analysis for distinguishing different Ki-67 expression levels was 0.711. It inferred that the CT images of lung adenocarcinoma were related to the expression of Ki-67 (30). Our results were consistent with previous findings (32–36). Thus, the conventional CT examination might indirectly reflect the proliferative activity of lung adenocarcinoma, which was of high value to the early identification of the positive Ki-67 expression from negative Ki-67 expression and the facilitation of early diagnoses and individualized treatments, improving the survival rate. However, the ability of CT images to predict the Ki-67 index is controversial. Conventional CT provides limited information regarding lung adenocarcinoma grading and cannot replace the biopsy and surgery in obtaining specimens for a definitive diagnosis (37).

Radiomics can extract information-rich imaging functions with high throughput, which is different from traditional subjective imaging, and can quantify imaging information that the human eye cannot detect (15, 38, 39). In mathematics, radiomic features have different functions and definitions. Thus, it has a very good advantage in measuring the heterogeneity of tumor texture features (40). Several studies have shown that radiomics have been effective in predicting the Ki-67 index in multiple tumors (41). In this study, we established a pre-operative Ki-67 classification model in patients with lung adenocarcinoma using CT-based radiomic features. The result shows that eight radiomic features were significantly different between the negative Ki-67 group and the positive Ki-67 group (*p* <0.001) (42). The CT-based radiomic predictive model demonstrated a stable and reliable performance, reaching an AUC of 0.871 and 0.8 and an accuracy of 80.6 and 75% in the training and testing cohorts, respectively. Therefore, the analysis revealed that CT-based radiomic features could pre-operatively predict Ki-67 levels in patients with lung adenocarcinoma, especially for suspicious patients under conservative treatment or patients who have lost the opportunity of a biopsy. The preliminary judgment of tumor proliferative activity through radiomic features can improve the accuracy and effectiveness of treatment, and could avoid the delay of disease and economic loss caused by ineffective treatment, which could have potential implications for future patient management and aid in the implementation of precision medicine.

Choosing an appropriate Ki-67 cut-off value is convenient for clinicians to treat and manage patients. However, no consensus on the prognostic value of the Ki-67 expression level was found among the published studies, neither according to disease stage nor histological subtype. In previous studies on lung cancer, the cutoff values for Ki-67 prediction of prognosis were mostly used at 25, 30, 40, and 50% (43–45). For stage I lung adenocarcinoma, a cut-off value of 0.1 was commonly used. Ishida suggested that the Ki-67 index of 2.8% might be used as a marker to distinguish between MIA and AIS (29). Determining a cut-off value is often based on the median value. In this study, 5% was selected as the classification threshold based on our data characteristics and previous similar studies, and relatively good results were obtained, which indicated that proliferative activity with a Ki-67 expression level of 5% may be a crucial turning point for progression from non-invasive adenocarcinomas (AAH/AIS/MIA) to invasive adenocarcinomas.

Our study had some limitations. First, this study had a small sample size, which may increase concerns regarding selection bias. Moreover, this study was a single-centered retrospective study. Further studies involving multiple centers and a large number of patients are necessary. Second, the manual outline of ROIs is time and labor-consuming, and there is no standardized outline process and rules, which may lead to poor consistency among different radiologists. The automatic recognition of tumor lesions and the characterization of ROIs for feature extraction are some of the future research directions. Third, the largest diameter of the lung adenocarcinoma lesions included in this study was <3 cm, and there is a bias in the selection of study subjects, which may affect the results of the study. In the future, how to better mine information to assist clinical decision-making so that patients can get more accurate individualized treatments is also an opportunity and challenge in the development of radiomics.

In conclusion, Ki-67 expression levels with a cut-off value of 5% could be used to differentiate non-invasive lung adenocarcinomas (AAH/AIS/MIA) from invasive lung adenocarcinomas. The radiomic characteristics of CT have potential as non-invasive biomarkers for predicting Ki-67 levels in patients with lung adenocarcinoma, which might allow for a precise evaluation of tumor biological behavior, aid in clinical treatment decision making for the precise management of patients with lung adenocarcinoma, as well as provide supplemental information for depicting the heterogeneity of lung adenocarcinoma in different histological subtypes.

## Data Availability Statement

The original contributions presented in the study are included in the article/supplementary material, further inquiries can be directed to the corresponding author/s.

## Ethics Statement

Written informed consent was obtained from the individual(s) for the publication of any potentially identifiable images or data included in this article.

## Author Contributions

ZH and ZX: conception and designation. ML and ZH: data collection. ML and ZA: data analysis and drafting the manuscript. ML and ZX: statistical analysis. YC and YL: technical support. All authors contributed to the article and approved the submitted version.

## Funding

This research was supported by grants from the National Natural Science Foundation of China (No. 82171931), Natural Science Foundation of Guangdong (No. 2015A030313753), the Science and Technology Program of Guangzhou (Nos. 201903010032 and 202102080572), and the Panyu Science and Technology Program of Guangzhou (Nos. 2017-Z04-12, 2019-Z04-01, and 2019-Z04-23).

## Conflict of Interest

The authors declare that the research was conducted in the absence of any commercial or financial relationships that could be construed as a potential conflict of interest. The reviewer ZW declared a shared affiliation with one of the authors, ML, to the handling editor at the time of the review.

## Publisher's Note

All claims expressed in this article are solely those of the authors and do not necessarily represent those of their affiliated organizations, or those of the publisher, the editors and the reviewers. Any product that may be evaluated in this article, or claim that may be made by its manufacturer, is not guaranteed or endorsed by the publisher.
